# Bisphosphonates in breast cancer radiotherapy: associations with skeletal complications and treatment outcomes - a retrospective cohort study

**DOI:** 10.3389/fonc.2026.1725699

**Published:** 2026-02-09

**Authors:** Khanom Baalbaki, Maya Hemdanieh, Jeffrey Yammine, Bshara Sleem, Souad Sawaf, Rawad Lakkis, Mohamad Nassereddine, Larry Bodgi

**Affiliations:** 1 Department of Orthopedics Surgery, American University of Beirut Medical Center, Beirut, Lebanon; 2 Faculty of Medicine, American University of Beirut, Beirut, Lebanon; 3 Department of Radiation Oncology, American University of Beirut Medical Center, Beirut, Lebanon; 4 Department of Anatomy, Cell Biology and Physiological Sciences, Faculty of Medicine, American University of Beirut, Beirut, Lebanon; 5 Unité INSERM UMR 1296 “Radiations: Défense, Santé, Environnement”, Lyon, France

**Keywords:** bisphosphonates, breast cancer, radiotherapy, skeletal complications, zoledronic acid

## Abstract

**Introduction:**

Breast cancer is the most diagnosed cancer in women worldwide. Radiotherapy is a cornerstone of treatment for both early-stage ductal carcinoma *in situ* (DCIS) and metastatic disease, but concerns about side effects remain. Bisphosphonates (BPs), particularly zoledronic acid (ZOL), have shown anti-tumor effects in preclinical models and reduce skeletal complications in patients with bone metastases. This study aimed to evaluate the radiosensitizing potential of BPs and their relationship with skeletal complications in breast cancer patients undergoing radiotherapy.

**Methods:**

We conducted a retrospective cohort study at the American University of Beirut Medical Center, including female patients aged 40–65 years with primary DCIS who received radiotherapy between November 2018 and September 2023. Data on patient demographics, tumor characteristics, treatment modalities, and outcomes were extracted from electronic medical records. Associations between BP use and pathologic fractures, musculoskeletal (MSK) complications, and treatment response were analyzed using logistic regression adjusted for relevant confounders.

**Results:**

Among 397 patients with breast cancer in our cohort, only 122 (30.7%) received ZOL. BP use was independently associated with increased odds of MSK complications (OR = 4.34; 95% CI: 2.42–7.77; p < 0.001) and pathologic fractures (OR = 2.93; 95% CI: 1.37–6.24; p = 0.005). No significant association was observed between BP use and tumor response, skin complications, fatigue, or other adverse effects.

**Conclusion:**

These findings likely reflect baseline skeletal fragility rather than a direct harmful effect of BPs. BPs remain an important indicator of bone vulnerability rather than a therapeutic modifier.

## Introduction

Breast cancer, the most prevalent diagnosed cancer in women globally, remains a significant public health burden despite advancements in diagnosis and treatment (1). Projected increases in diagnosed cases and deaths by 2040 highlight the urgency for continued research and refinement of our approach to managing this diverse disease (2). Understanding breast cancer’s complexity requires a nuanced classification system. While histopathological staging remains the gold standard, the disease encompasses various subtypes within two main categories: *in situ* and infiltrative carcinoma (3). Notably, Ductal Carcinoma in Situ (DCIS), accounting for 25% of diagnoses, ranges from low grade lesions to highly aggressive forms (4). Radiotherapy plays a crucial role in both early and advanced stages. In early-stage DCIS, it reduces recurrence by 50% regardless of tumor characteristics (5). For patients with bone metastases, a common complication in stage IV, radiotherapy alleviates pain, prevents fractures, and controls bone remodeling (6, 7). However, concerns about side effects have led to a trend towards de-escalating therapy in DCIS management (8). While national guidelines permit excluding radiotherapy after lumpectomy for low-risk cases, identifying these cases remains challenging (9, 10). Additionally, mortality from breast cancer after DCIS treatment is minimal regardless of the method, meaning omitting radiotherapy could increase recurrence risk (11). This highlights the need for agents that maximize radiotherapy benefits while minimizing side effects. One promising avenue lies in bisphosphonates (BPs), bone-modifying agents with proven efficacy in both early and metastatic breast cancer (12–14). BPs as zoledroinic acid exhibit anti-tumor effects through apoptosis induction, angiogenesis inhibition, and immune stimulation (15–18). Additionally, they prevent skeletal complications in patients with bone metastases (19). Combining BPs with radiotherapy holds particular promise. Studies like the *in-vitro* one by Ural et al. (20) demonstrate the synergistic effect of zoledronic acid and radiotherapy on breast cancer cells. Furthermore, Restier-Verlet et al. (21) showed the radioprotective potential of combining zoledronic acid with statins for normal cells. These findings suggest that BPs, with their dual effect of radiosensitization and radioprotection, might be superior to other agents. However, limited studies have shown their clinical effect as radiosensitizers in breast cancer, whether in early stage or locally advanced stage. Building on existing evidence, this study aims to evaluate the radiosensitizing potential of bisphosphonates, particularly zoledronic acid, in breast cancer treatment, and to investigate their relationship with skeletal complications in patients undergoing radiotherapy.

## Materials and methods

### Study design and setting

This is a retrospective cohort study conducted at the American University of Beirut Medical Center (AUBMC), a tertiary care academic hospital in Lebanon. The study included all female patients between the ages of 40 and 65 years who were diagnosed with primary breast cancer (pathology biopsy proven ductal carcinoma *in situ*, DCIS) and received radiation therapy at the Department of Radiation Oncology at AUBMC between November 2018 and September 2023. The study was approved by the Institutional Review Board (IRB) at AUBMC under the protocol number (BIO-2024-0042). Informed consent was waived given the retrospective nature of the study. To protect patient confidentiality, identifying information such as names was not collected. Each patient was assigned a unique study ID that was stored on a separate log sheet accessible only to the principal investigator and the research coordinator.

### Study population

The study population consisted of female patients aged 40–65 years with a confirmed diagnosis of breast cancer, including ductal carcinoma *in situ* (DCIS), invasive, and metastatic disease, who underwent radiation therapy at AUBMC during the study period. Patients who had a prior history of invasive breast cancer in the ipsilateral breast were excluded.

### Data collection and sampling

Eligible patients were identified through the hospital’s electronic medical record system (Epic System). Medical charts and pathology reports were reviewed to extract the required data. Data were retrieved and entered into a secure Excel spreadsheet. A coding system was applied to ensure consistency and facilitate data management and analysis. The data collection sheet was divided into sections that included patient demographics and medical history (age at diagnosis, smoking status, family history, and comorbidities), tumor characteristics (pathology, staging, ER/PR/HER2 status), treatment modalities (radiotherapy, chemotherapy, hormone therapy, and bisphosphonate use), and outcomes (FRAX score, musculoskeletal complications, post-radiotherapy pathologic fractures, and other complications).

### Statistical analysis

Data analysis was performed using IBM SPSS Statistics software, version. Descriptive statistics were used to summarize patient demographics, comorbidities, tumor characteristics, and treatment modalities. Continuous variables were reported as means with standard deviations or medians with interquartile ranges, as appropriate. Categorical variables were reported as frequencies and percentages. Comparative analyses were conducted to evaluate the effect of bisphosphonate use on treatment outcomes and skeletal complications. Associations between categorical variables were assessed using the chi-square test. A p-value of <0.05 was considered statistically significant.

## Results

A total of 397 breast cancer patients who received radiotherapy were included in the analysis. Among them, 124 patients (31.2%) were treated with a bisphosphonate, specifically zoledronic acid (ZOL), while 273 patients (68.8%) did not receive bisphosphonate therapy. Baseline demographic, clinical, and tumor characteristics of the study population, stratified by bisphosphonate use, are summarized in **Table 1**. The median radiotherapy dose was 42.6 Gray (range 0–504 Gray), with a median of 16 treatment sessions (range 0–130).

**Table 1:** Baseline characteristics of breast cancer patients receiving radiotherapy, stratified by bisphosphonate use.

Characteristic	No bisphosphonates	Zoledronic acid
Number of patients	273	124
Age at diagnosis, years	49.6 ± 8.7	50.7 ± 9.4
Family history of breast cancer	103 (37.7%)	39 (31.5%)
Any comorbid illness	108 (39.6%)	55 (44.4%)
Hypertension	57 (20.9%)	34 (27.4%)
Diabetes mellitus	30 (11.0%)	23 (18.5%)
Dyslipidemia	48 (17.6%)	26 (21.0%)
Cardiovascular disease	38 (13.9%)	23 (18.5%)
Cancer stage		
DCIS	27 (10.0%)	3 (2.5%)
Stage I	69 (25.6%)	16 (13.1%)
Stage II	100 (37.0%)	24 (19.7%)
Stage III	41 (15.2%)	22 (18.0%)
Stage IV	33 (12.2%)	57 (46.7%)
Estrogen receptor (ER) positive	207 (76.4%)	106 (85.5%)
Progesterone receptor (PR) positive	176 (64.9%)	89 (71.8%)
Human Epidermal Growth Factor Receptor 2(HER2) positive	102 (37.6%)	44 (35.5%)

With respect to disease stage, patients who did not receive bisphosphonates were more frequently diagnosed with early-stage disease, including DCIS (10.0%), stage I (25.6%), and stage II (37.0%). In contrast, patients treated with zoledronic acid demonstrated a higher burden of advanced disease, with stage III and stage IV cancers accounting for 18.0% and 46.7% of cases, respectively. Notably, stage IV disease was substantially more prevalent in the zoledronic acid group compared with patients who did not receive bisphosphonates (46.7% vs. 12.2%). Most tumors were estrogen receptor (ER)-positive (76.4%), progesterone receptor (PR)-positive (64.9%), and 37.6% were Human Epidermal Growth Factor Receptor 2 (HER2) -positive.

Absolute event counts (n/N, %) for clinical outcomes stratified by bisphosphonate exposure are presented in **Table 2**. Patients receiving zoledronic acid experienced higher rates of musculoskeletal complications, pathologic fractures, and mortality compared with those who did not receive bisphosphonates.

**Table 2:** Clinical outcomes according to bisphosphonate (zoledronic acid) use in patients receiving radiotherapy.

Outcome	No bisphosphonates	Zoledronic acid
Tumor response*		
Progression	102/258 (39.5%)	55/112 (49.1%)
Regression	109/258 (42.2%)	31/112 (27.7%)
Cure	47/258 (18.2%)	26/112 (23.2%)
Breast pain	25/273 (9.2%)	20/124 (16.1%)
Skin complications	152/273 (55.7%)	54/124 (43.5%)
Pathologic fracture	20/271 (7.4%)	30/124 (24.2%)
Fatigue	18/273 (6.6%)	12/124 (9.7%)
Other complications	8/273 (2.9%)	2/124 (1.6%)
Musculoskeletal complication	46/272 (16.9%)	52/124 (41.9%)
Death	23/273 (8.4%)	31/124 (25.0%)
Any overall side effect	195/273 (71.4%)	97/124 (78.2%)

*Values are presented as mean ± standard deviation or number (%), as indicated, with percentages calculated within bisphosphonate exposure groups. Analyses were restricted to patients who received radiotherapy. Estrogen receptor (ER), progesterone receptor (PR), and HER2 status were available for 395 patients.

### Unadjusted analyses

In unadjusted analyses, zoledronic acid (ZOL) use was significantly associated with tumor response (χ² = 7.06, df = 2, p = 0.029), pathologic fractures (χ² = 21.75, df = 1, p < 0.001), musculoskeletal (MSK) complications (χ² = 28.64, df = 1, p < 0.001), skin complications (χ² = 5.03, df = 1, p = 0.025), and breast pain (χ² = 4.12, df = 1, p = 0.042). No significant associations were found with fatigue, other complications, or overall side effects.

### Adjusted analyses

All logistic regression analyses were adjusted for age at diagnosis, smoking status, comorbid illness, staging, ER, PR, Her2, hormone therapy, bisphosphonate use, and chemotherapy.

#### Musculoskeletal complications

When compared with patients not taking a bisphosphonate, ZOL use was strongly associated with increased odds of musculoskeletal complications (odds ratio = 4.34; 95% confidence interval: 2.42–7.77; p < 0.001). In other words, patients who received bisphosphonates were more than four times as likely to experience musculoskeletal problems, such as bone or joint issues.

Estrogen receptor (ER) positivity was also significantly associated with these complications (OR = 2.44; 95% CI: 1.08–5.51; p = 0.031), indicating that patients with ER-positive breast cancer had roughly twice the risk compared to those who were ER-negative. Patients who did not receive hormone therapy had a lower chance of developing musculoskeletal complications; about half the risk compared to those who did receive hormone therapy (OR = 0.48; 95% CI: 0.25–0.93; p = 0.029).

Chemotherapy showed a trend toward higher risk (OR = 2.18; 95% CI: 0.93–5.11; p = 0.073); however, this association did not reach statistical significance.

#### Pathologic fractures

Pathologic fractures occurred in 49 of 305 patients (16.1%) who received chemotherapy compared with 1 of 90 patients (1.1%) who did not receive chemotherapy. Pathologic fractures were significantly associated with bisphosphonate use (OR = 2.93; 95% CI: 1.37–6.24; p = 0.005), chemotherapy (OR = 14.58; 95% CI: 1.74–122.24; p = 0.013), absence of hormone therapy (OR = 0.34; 95% CI: 0.15–0.80; p = 0.014), and older age (OR = 1.05 per year; 95% CI: 1.01–1.09; p = 0.011).

#### Skin complications

No variables, including bisphosphonates (ZOL) or chemotherapy, were significantly associated with skin complications (all p > 0.05).

#### Breast pain

Breast pain was associated with age (OR = 1.04 per year; 95% CI: 1.00–1.08; p = 0.039), while ZOL and other covariates were not significant (p > 0.05).

#### Fatigue

No significant associations were observed between bisphosphonate use, chemotherapy, or other covariates and reported fatigue (p > 0.05).

#### Other complications

Bisphosphonate use and chemotherapy were not significantly associated with other complications (p > 0.05).

#### Tumor response

For tumor response, nominal regression indicated no association with bisphosphonate use (p = 0.238); however, comorbid illness (p = 0.015) and staging (p = 0.070) were significant factors.

Overall, bisphosphonate use was independently associated with a markedly increased risk of musculoskeletal complications and pathologic fractures, but not with tumor response or most other complications after adjustment.

## Discussion

Our study was designed to evaluate whether BPs in particular ZOL could enhance radiosensitization of tumors and reduce skeletal complications in breast cancer patients receiving radiotherapy. We aimed to determine if bisphosphonates help radiosensitize tumors and reduce bone complications in breast cancer patients getting radiotherapy. Contrary to early laboratory hypotheses, our findings revealed no radiosensitizing benefit. Instead, we observed a higher incidence of pathologic fractures and musculoskeletal (MSK) complications in patients receiving zoledronic acid. Although baseline bone mineral density (BMD) data were not available, the higher incidence of fractures and musculoskeletal complications among patients receiving BPs may reflect underlying skeletal vulnerability rather than a direct harmful effect of bisphosphonates.

Laboratory studies have revealed promising radiosensitizing effects of BPs—for example, *in vitro* pre-treatment with zoledronic acid combined with pravastatin (ZOPRA) decreased radio resistance in breast cancer cell lines, suggesting impaired DNA repair processes (22). In preclinical breast cancer models, BPs have shown both direct and indirect anti-tumor effects. *In vitro*, they inhibit tumor cell adhesion, invasion, proliferation, and induce apoptosis (23, 24). *In vivo*, BPs modify the bone microenvironment reducing osteolysis, limiting bone-derived growth factors, and exerting antiangiogenic and immunomodulatory effects (25, 26). There is emerging evidence that certain bisphosphonates may enhance radiosensitivity in breast cancer cells (22, 27). In another instance, Kim et al. demonstrated that zoledronic acid (ZOL) increased DNA damage and reduced survival in irradiated osteosarcoma cells, suggesting a potential radiosensitizing effect through impaired DNA repair pathways [8]. Similarly, in breast cancer cell models, ZOL combined with radiation led to synergistic growth inhibition.

Nevertheless, clinical outcomes remain inconsistent. Some adjuvant trials suggested reduced recurrence and mortality among postmenopausal women treated with bisphosphonates (28, 29). Furthermore, a Japanese study by Tanaka et al. showed that combination of radiotherapy and BPs have shown significantly better tumor response and survival compared to BPs alone in patients with osteolytic metastases (30). This suggests a role for synergy, even if not directly linked to radiosensitization in the strictest sense. The Early Breast Cancer Trialists’ Collaborative Group (EBCTCG) meta-analysis showed that adjuvant bisphosphonates reduced bone recurrence and breast cancer mortality only in postmenopausal women, with no significant effect in premenopausal women (31). The updated American Society of Clinical Oncology-Ontario Health (ASCO-OH) guidelines similarly recommend adjuvant bisphosphonates for postmenopausal patients but emphasize modest benefit and advocate against denosumab in this context due to inconsistent recurrence reduction (32). However, other studies like the one by Scafetta et al. (2025) (33) did not confirm a general radiosensitizing benefit, which align with our findings.

Although zoledronic acid use was correlated with more pathologic fractures in our cohort, this is most likely explained by confounding factors: patients with heightened fracture risk attributed to bone metastases, osteoporosis, or advanced disease are preferentially prescribed BPs. Therefore, the association does not imply that BPs cause fractures; rather, they are markers of worse baseline bone health. This interpretation aligns with the intended use of BPs to prevent fractures in high-risk populations.

Indeed, confounding by indication likely drives the association with pathologic fractures. BPs are preferentially prescribed to patients with advanced disease—those already at higher risk for skeletal-related events—making the observed fracture risk more an indicator of baseline fragility than drug-induced harm. Similarly, musculoskeletal complications such as bone pain or stiffness are known adverse effects of BPs and are often encountered in patients with compromised bone integrity (34, 35).

Beyond these specified outcomes, other analyses revealed that BP use was not significantly related to tumor response, breast pain, skin complications, fatigue, other complications, or overall side effects in adjusted models. This suggests that initial associations could be attributable to underlying patient or disease characteristics rather than medication effects.

Preclinical models support anti-cancer and radiosensitizing potential of BPs, but these findings do not uniformly translate to clinical benefit. In controlled settings, BPs are not prescribed for direct tumor targeting but for skeletal support, a nuance that must be emphasized. Observational studies suggest possible recurrence or metastasis risk reduction in postmenopausal women (22, 26, 36, 37); however, randomized trials have yielded mixed results. For instance, adjuvant zoledronic acid showed benefits mainly in postmenopausal patients, while others found no significant overall survival advantage (38). Additionally, some observational associations between BPs and reduced breast cancer risk were later challenged by controlled trials, underscoring confounding effects (39–41).

In clinical practice, international guidelines such as those from ASCO (American Society of Clinical Oncology) and ESMO (European Society for Medical Oncology) recommend the use of bisphosphonates primarily for the prevention of skeletal-related events in patients with breast cancer and bone metastases, not as tumor-directed agents (32, 42). Our findings are consistent with this, as no radiosensitizing or tumor protective effect was demonstrated. While BP use was associated with increased pathological fractures and musculoskeletal complications, this more likely reflects baseline patients’ risk rather than casual side effect or harm.

Lastly, our study reinforces current guideline-based practice: bisphosphonates remain an important supportive therapy to reduce skeletal morbidity, but there is insufficient evidence to justify their use as radiosensitizers in breast cancer. Future prospective trials with standardized RT and different BPs are needed to clarify potential interactions and explore mechanistic insights suggested by preclinical data.

## Conclusion

In conclusion, BP use was associated with increased pathologic fractures and musculoskeletal complications, but these findings likely reflect baseline patient risk rather than causal harm. No clear tumor-protective or radiosensitizing effect of BPs was demonstrated in our study; however, their use remains an important for skeletal fragility rather than a therapeutic modifier.

These results underscore the importance of recognizing BP prescription as a marker for underlying bone vulnerability. Future prospective, multicenter trials with standardized treatment protocols and comprehensive bone health assessments are needed to definitively clarify the role of bisphosphonates in enhancing radiotherapy outcomes for breast cancer.

### Limitations

Our study has several limitations that warrant consideration. First, its retrospective observational design is inherently subject to confounding, particularly confounding by indication, which likely explains the observed associations between BP use and both pathologic fractures and musculoskeletal complications. Additionally, our analysis focused on a single BP, zoledronic acid. Although we adjusted for several potential confounders, residual confounding from unmeasured variables, as baseline bone mineral density, duration and dose of BP therapy, or severity of bone metastasis, cannot be excluded. Finally, the single-center nature of the study may limit generalizability, and prospective multicenter trials with standardized treatment protocols are needed to validate these findings.

## Data Availability

The data analyzed in this study is subject to the following licenses/restrictions: The data was extracted from medical record system(epic) and it is restricted to the hospital. Requests to access these datasets should be directed to Dr Mohamad Nasseredine(the corresponding author): mn103@aub.edu.lb.
